# β-Catenin nuclear localization positively feeds back on EGF/EGFR-attenuated AJAP1 expression in breast cancer

**DOI:** 10.1186/s13046-019-1252-6

**Published:** 2019-06-06

**Authors:** Cong Xu, Fang Liu, Guomin Xiang, Lu Cao, Shuling Wang, Jing Liu, Qingxiang Meng, Danni Xu, Shuhua Lv, Jiao Jiao, Yun Niu

**Affiliations:** 1 Tianjin Medical University Cancer Institute and Hospital, National Clinical Research Center of Cancer, Key Laboratory of Cancer Prevention and Therapy, Tianjin, Tianjin’s Clinical Research Center for Cancer, Tianjin, 300060 China; 2 Key Laboratory of Breast Cancer Prevention and Therapy, Tianjin Medical University, Ministry of Education, West Huanhu Road, Ti Yuan Bei, Hexi District, Tianjin, 300060 Tianjin China; 3 Department of Breast Cancer Pathology and Research Laboratory, Tianjin Medical University Cancer Institute and Hospital, Tianjin, China; 4 Department of Breast Oncology, Tianjin Medical University Cancer Institute and Hospital, Tianjin, China; 5 Department of Pathology, Tianjin Union Medical Center, Tianjin People’s Hospital, Tianjin, 300121 China

**Keywords:** AJAP1, β-Catenin, Nuclear location, EGF, EGFR, Tumor progression

## Abstract

**Background:**

Adherent junction associated protein 1 (AJAP1), a typical molecule of adherent junctions, has been found to be a tumor suppressor in many cancer types. Aberrant activation of β-catenin has been demonstrated to be associated with malignant biological properties of tumors including breast cancer. This study aimed to investigate the function and mechanism of AJAP1-mediated β-catenin activity of breast cancer lines in vitro and in breast cancer patients.

**Methods:**

AJAP1 and β-catenin expressions in breast cancer tissues and cell lines were detected by immunohistochemistry, western blotting and qRT-PCR. The EGF/EGFR axis-mediated AJAP1 attenuated β-catenin nuclear location was measured by western blotting, immunofluorescence assay, co-immunoprecipitation, luciferase assay and ubiquitination assays. Furthermore, the function of AJAP1 and β-catenin regulated breast cancer progression was explored both in vivo and in vitro*.*

**Results:**

It was found that AJAP1 had a high negative correlation with β-catenin nuclear expression and was a novel tumor suppressor in breast cancer. AJAP1 loss can mediate β-catenin accumulated in cytoplasm and then transferred it to the nucleus, activating β-catenin transcriptional activity and downstream genes. Additionally, β-catenin can reverse the invasion, proliferation ability and tumorigenicity of the depletion of AJAP1 caused both in vivo and in vitro. Besides, EGF/EGFR also involved in the process of AJAP1-depiction induced β-catenin transactivation to the nucleus. More importantly, EGFR depletion/AJAP1 knocked down promoted the progression of breast cancer by regulating the activity of β-catenin nuclear transactivation.

**Conclusion:**

This study demonstrated that AJAP1 acted as a putative tumor suppressor while β-catenin nuclear localization positively fed back on EGF/EGFR-attenuated AJAP1 expression in breast cancer, which might be beneficial to develop new therapeutic targets for decreasing nuclear β-catenin-mediated malignancy in breast cancer.

**Electronic supplementary material:**

The online version of this article (10.1186/s13046-019-1252-6) contains supplementary material, which is available to authorized users.

## Background

Breast cancer, a biologically and molecularly heterogeneous disease derived from epithelial cells, has been one of the most common malignancies in women worldwide for many years [1–3]. As fundamental components of epithelial cells, adherent junctions (AJs) have been proven to play important roles in cancer progression [4–10]. However, data on AJs in breast cancer is still scarce.

Adherens junctions-associated protein 1(AJAP1), also called Shrew-1, was initially discovered as a novel transmembrane protein of AJs in epithelial cells [11]. Some studies then verified that AJAP1 was a promising tumor candidate gene in glioma [12, 13], hepatocellular carcinoma [14–16], esophagus carcinoma [17] and oligodendrogliomas [18]. However, its role in breast cancer has not been fully elucidated.

In addition, previous reports showed that 50% of breast cancer cases have Wnt signaling abnormal activation and low rates of somatic mutations [19–21]. Additionally, abnormal activation of Wnt signaling often led to β-catenin nuclear accumulation [22–25]. Nuclear β-catenin can function as a transcriptional co-activator of the TCF/LEF complex, resulting in a series of changes in proliferation, invasion and metastasis. Moreover, β-catenin has been implicated in the transduction of mechanical signals from junctions to the nucleus [26].

In this study, the roles of AJAP1 and β-catenin in breast cancer were explored. Immunohistochemistry assay showed that AJAP1 depletion was positively related with β-catenin nuclear expression and poor prognosis of patients. Besides, AJAP1 was a putative tumor suppressor that suppressed the growth, migration, invasion of breast cancer and cell cycle by mediating the nuclear β-catenin activity. More importantly, β-catenin localization and tumor progression also positively fed back on EGF/EGFR-attenuated AJAP1 expression. In summary, these findings might be beneficial in developing new therapeutic targets for decreasing nuclear β-catenin-mediated malignancy in breast cancer.

## Materials and methods

### Patients and breast cancer samples

283 cases of paraffin-embedded breast cancer patients’ specimen and 25 pairs of fresh tumor tissues were randomly selected at Cancer Hospital of Tianjin Medical University. The patients received treatments from January 1, 2006 to December 31, 2006. None of the patients underwent chemotherapy or radiotherapy before surgery. The patient clinical pathologic features are showed in Additional file 1: Table S1. All cases had decent follow-up and reliable clinical data. Besides, this study followed the Declaration of Helsinki, and the patients provided written informed consents.

### Immunohistochemistry (IHC) and evaluation

All paraffinized tissue blocks were cut at 4 μm thicknesses and detected by the SP immunochemistry kit (Zhongshan Golden Bridge Biotechnology, Beijing, China). IHC assay was conducted as previously described [27]. The rabbit monoclonal anti-human AJAP1 antibody (Bioss, China) at 1:100 dilution or the mouse monoclonal anti-human β-catenin antibody (CTNNB1, Boster) at 1:200 dilution was used for IHC. Two senior pathologists (Yun Niu and Shuhua Lv) evaluated the score without any knowledge of the clinicopathological outcomes of the patients. The percentage of positivity of the tumor was scored as “0” (no tumor cells), “1” (1–25%), “2” (26–50%), “3” (51–75%), and “4” (> 75%). The staining intensity of the positive tumor cells was scored as “0” (no staining), “1” (weak staining), “2” (moderate staining), and “3” (strong staining). As for AJAP1, the multiplier of the scoring of (0–3) for low expression and (4–12) for high expression were used. The IHC staining results of β-catenin were evaluated independently according to the subcellular localization of the nucleus and membrane. A positive/abnormal nuclear expression was defined as over 10% of the nuclear-stained tumor cells. An abnormal membranous expression demonstrated either no immunoreactivity in the membrane or less than 10% of the cancer cells with a positive membranous staining [28, 29].

### Cell line and culture

All cell lines were purchased from the American Type Culture Collection (ATCC, USA). T47D, SK-BR-3, MCF-7 and MDA-MB-231 were cultured with the 1640 (Gibco, USA), MCF10A was cultured in DMEM-F12 (Gibco, USA) media. MDA-MB-453 was cultured in L-15 media. All media contained 10% fetal bovine serum (FBS) and 1% penicillin/streptomycin and cell culture carried out at 37 °C in a 5% CO_2_ incubator.

### Immunofluorescence

The cells were grown in 24 -well plates and then stimulated with 50 ng/ml EGF for 24 h. After 1 day, the cells were fixed, rehydrated, and incubated with β-catenin (Santa Cruz Biotech, China), AJAP1 (Bioss, China), and fluorescein-labeled secondary antibodies. The nuclei were stained with DAPI for 10 min. The slides were examined through a fluorescent confocal microscope.

### Western blot

Cellular protein was extracted by RIPA buffer containing PMSF. The Nuclear and Cytoplasmic Isolation Kit (KeyGEN, China) was used for the cytoplasmic and nuclear protein extraction according to the manufacturer’s instructions. The proteins were separated on 10% SDS-PAGE gels, transferred onto PVDF membranes, blocked using 5% skim milk, and incubated with primary antibodies overnight at 4 °C. The protein bands were detected by the ECL detection Kit (Solarbio, China).

### Plasmids and lentiviral transfection

The related siRNAs and control plasmids were showed in Additional file 2: Table S2. The viruses were packaged in 293 T cells according to the manufacturer’s instructions. The infected cells were selected with 2 μg/mL puromycin.

### Quantitative reverse-transcription polymerase chain reaction (qRT- PCR) assay

Total RNAs were extracted using Trizol reagent (Takara, Japan). Reverse transcription was conducted by the SuperScript RT kit (Takara, Japan). The qRT-PCR assay was performed with SYBR Green PCR kit (Takara, Japan). Primers used are listed in Additional file 2: Table S2. GAPDH was used as the internal control. The relative expression levels were calculated by the 2^-ΔΔCt^ method. All experiments were assayed in triplicate.

### Co-immunoprecipitation (co-IP)

The co-IP of AJAP1 and β-catenin was performed using the Pierce Co-IP kit (Thermo Scientific, USA) according to the manufacturer’s protocol as described previously [30]. The protein quantity was then detected by western blotting.

### Luciferase assays

5 × 10^4^ cells were seeded in 24-well plates and cultured for 24 h at a density of 70–80%. The cells were transferred with 2 μg of β-catenin-responsive firefly luciferase reporter plasmid TOP-FLASH or the negative control FOP-FLASH (Merck-Millipore) using fugene6 (Invitrogen, USA) according to the manufacturer’s instructions. The cells were subjected to luciferase reporter assay 24 h after transfection according the Dual Luciferase Reporter Assay System (Promega, Madison, WI). The relative Renilla luciferase activity was normalized to that of firefly luciferase activity. Each experiment was measured in triplicate.

### Migration and invasion assay

2 × 10^4^ cells were seeded in the upper chamber with RMPI 1640 medium without FBS and a medium containing 10% FBS at 37 °C in the lower chamber. After culturing at 37 °C for 24 h, the cells in the upper chamber were harvested and then stained according to the three-step set (Thermo Scientific, USA) protocol. Scratches were made when the cells reached a 95% confluence in each well using a 10 μL plastic pipette tip. The scratches were measured and photographed. Each experiment was conducted at least three times.

### MTT and colony-formation assay

2 × 10^3^ cells were seeded in 96-well plates and the activities of the viable cells were measured at different time points using a microplate reader. In the colony formation assay, 8 × 10^2^ cells were cultured in 6-well plates for 2 weeks. The colonies were stained with crystal violet and counted.

### Subcutaneous xenograft assay

Female BALB/c nude mice (4–6 week) were injected with 5 × 10^6^ treated cells and kept in the Tianjin Medical University Cancer Institute and Hospital SPF’s animal feeding center. The tumors were measured as described previously [31]. All animals were sacrificed after 60 days. All experimental procedures were approved by the International Animal Care and Use Committee of Tianjin Medical University Cancer Institute and Hospital.

### Statistical analyses

Statistical significance was determined by SPSS version 24.0. Values are shown as mean ± SD. Multiple groups were compared by one-way analysis of variance followed by Dunnett’s t-test. *p* < 0.05 was considered statistically significant.

## Results

### AJAP1 was low expressed in breast cancer and inhibited the proliferation of breast cells

**Fig. 1:**
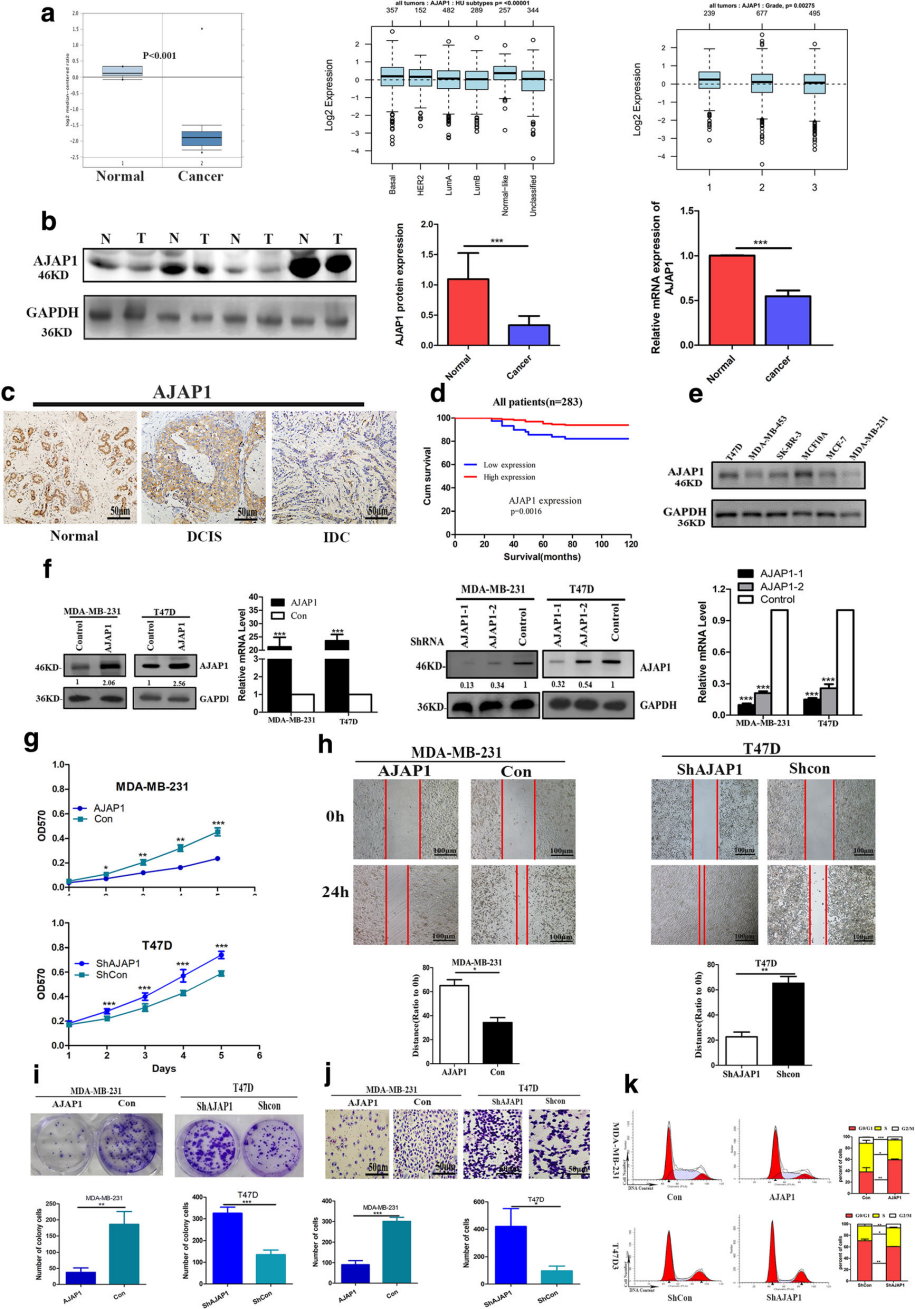
AJAP1 was low expressed in breast cancer and inhibited the proliferation of breast cells. **a** Dataset from Oncomine and GOBO on AJAP1 in tumors tissues versus non-tumor tissues (*n* = 59), different subtypes of breast cancer(*n* = 1881) and grades(*n* = 1411). **b** AJAP1 protein and mRNA levels in 25 pairs of tumor tissues versus non-normal tissue. **c** Representative IHC image of AJAP1 expression location in different stages of breast cancer (normal lobules, Ductal carcinoma in situ, invasive cancer). **d** overall survival of AJAP1 in 283 cases of breast cancer. **e** AJAP1 protein levels in breast cancer cell lines. **f** confirmation of upregulation and downregulation of AJAP1 expression by Western blot and qRT-PCR (**g-k**) effects of AJAP1 knockdown and overexpressed on in vitro proliferation (**g, i**), invade (**h**) migration(**j**) and cell cycle progression (**k**) Data were shown as mean ± SD *p < 0.05; **p < 0.01; ***p < 0.001

To determine the expression profile of AJAP1 in breast cancer tissues, breast cancer datasets from Oncomine and GOBO datasets were analyzed in this study. The dataset from Finak (GOBO)and Oncomine showed that AJAP1 was downregulated in the breast cancer tissues compared with the normal tissues (Fig. 1a left panel). In addition, GOBO dataset demonstrated that the expression of AJAP1 was related with cancer subtypes (Fig. 1a middle panel, *p* = 0.00402) and grades (Fig. 1a right panel, *p* = 0.00275). These conclusions were confirmed by Western blotting and qRT-PCR of 25 pairs of breast cancer and corresponding normal tissues (Fig. 1b).

Furthermore, 283 breast cancer formalin-fixed, paraffin-embedded tumor slides with complete clinicopathological characteristics and follow-up data were utilized to analyze the expression of AJAP1. In this work, AJAP1 was mainly located in the cytoplasm and was downregulated in breast cancer. Moreover, its expression was decreased in the progression of breast cancer (Fig. 1c). As shown in Table 1, low AJAP1 expression was significantly associated with advanced histological grade and higher lymph node involvement. In addition, Cox proportional hazards regression analyses (Additional file 3: Table S3) demonstrated that AJAP1 was an independent prognostic marker for overall survival (*p* = 0.024). Meanwhile, Kaplan-Meier analysis showed that patients with low expression of AJAP1 exhibited poor survival (Fig. 1d).

**Table 1:** The clinicopathologic characteristics of AJAP1 expression in 283 Breast cancer patients

Parameters	Cases	AJAP1		*p*
		Low expression (N, %)	High expression (N, %)	
Age(yr)		118(41.7)	165(58.3)	
≤50	130	49(41.5)	81(49.1)	0.208
> 50	153	69(58,5)	84(50.9)	
Menopausal status				
Premenopausal	156	60(50.8)	96(58.2)	0.221
Postmenopausal	127	58(49.2)	69(41.8)	
Family history				
No	194	85(72.0)	109(66.1)	0.286
Yes	89	33(28.0)	56(33.9)	
Tumor size				
T1	103	42(35.6)	68(41.2)	0.615
T2	148	66(55.9)	83(50.3)	
T3	32	10(8.5)	14(8.5))	
Histological grade				
1	44	9(7.6)	35(21.2)	< 0.0001*
2	153	59(50.0)	94(57.0)	
3	86	50(42.4)	36(21.8)	
LN involvement				
0	180	65(55.1)	115(69.7)	0.010*
1–3	43	19(16.1)	24(14.5)	
4–9	41	20(16.9)	21(12.7)	
≥ 10	19	14(11.9)	5(3.0)	
ER				
Negative	111	50(42.4)	61(37)	0.359
Positive	172	68(57.6)	104(63)	
PR				
Negative	142	83(41.5)	82(49.7)	0.849
Positive	141	35(42.2)	83(50.3)	
Her-2				
Negative	200	83(41.5)	117(58.5)	0.917
Positive	83	35(42.2)	48(57.8)	
Ki67				
< 20	83	31(26.3)	52(31.5)	0.339
≥ 20	200	87(73.7)	113(68.5)	
P53				
Negative	143	59(50)	84(50.9)	0.880
Positive	140	59(50)	81(49.1)	

*significantly different

To better explore the role of AJAP1 in breast cancer, AJAP1 expression in different wild types of breast cancer cell lines were detected. T47D and MDA-MB-231 cells showed typical high and low expression of AJAP1, respectively (Fig. 1e). T47D cells with knocking down of AJAP1 expression and MDA-MB-231 cells with overexpression of AJAP1 were then generated (Fig. 1f) to evaluate the proliferation, migration and invasion of breast cancer by MTT, Transwell, colony-formation and wound-healing assays. These results showed that AJAP1-depletion in T47D cells promoted the capacity of proliferation (Fig. 1g, Fig. 1i), migration (Fig. 1h) and invasion (Fig. 1j). In contrast, AJAP1 overexpressed in MDA-MB-231 cells showed the opposite results. Furthermore, flow cytometry (Fig. 1k) results showed that downregulation of AJAP1 in T47D cells significantly reduced the population of G0/G1 cells but increased S/G2/M cells. On the contrary, AJAP1-overexpressed MDA-MB-231 cells showed the opposite results. In conclusion, AJAP1 was a putative tumor suppressor in breast cancer.

### Different expression patterns of AJAP1 and β-catenin in breast cancer patients

**Fig. 2:**
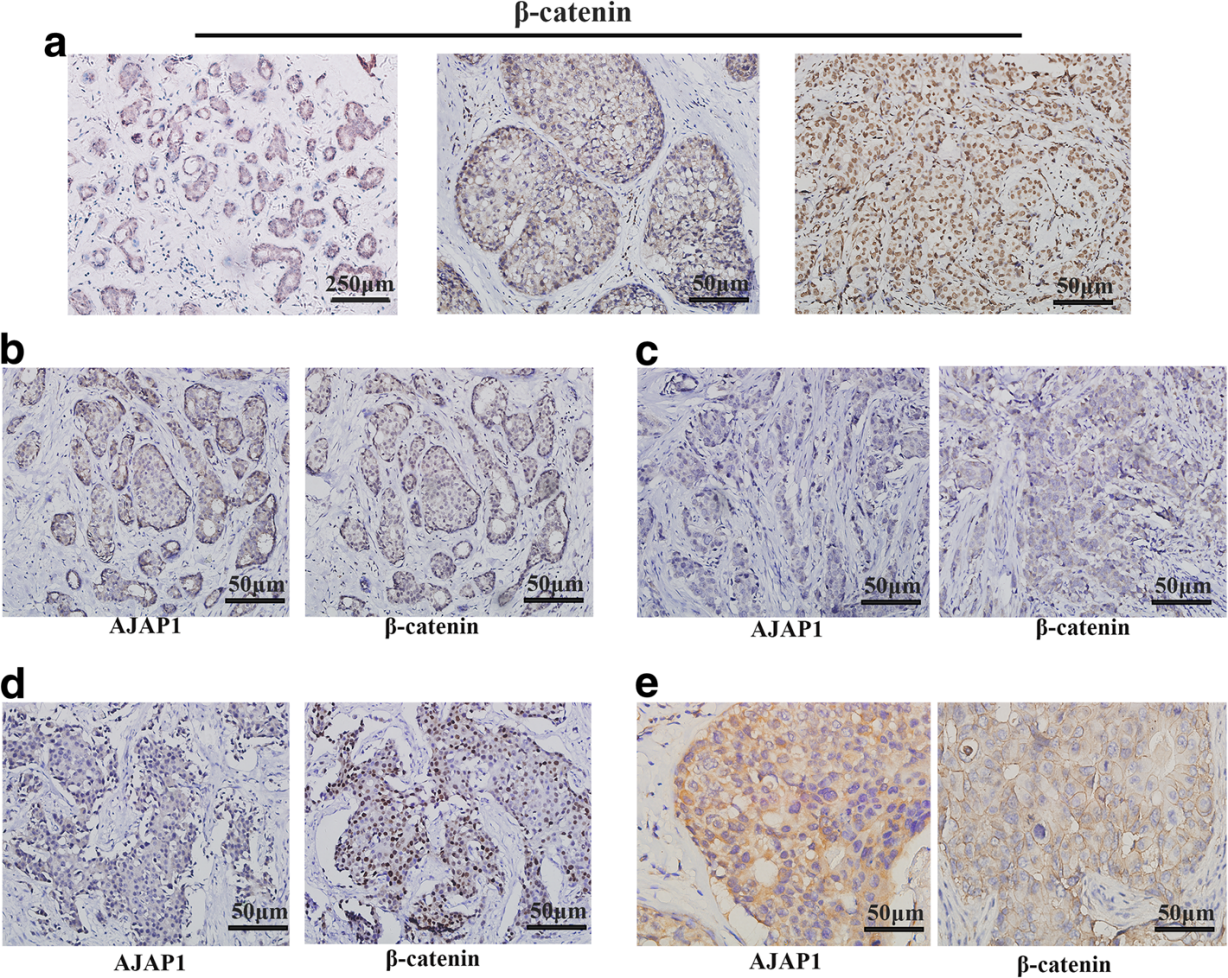
Immunohistochemical expression of β-catenin and AJAP1 in breast tissue slides. **a** β-catenin expression in normal breast cancer tissues, ductal carcinoma in situ and invasive ductal carcinoma (magnification, × 40, × 200 and × 200). **b-e** Different expression patterns of AJAP1 and β-catenin in breast cancer tissues. Micrographs showing low expression (**c, d**) and high expression (**b, e**) AJAP1 cytoplasm (**b, c, d**) and membrane (**e**) expression and negative (**c**) and positive (**b, d, e**) β-catenin membrane(**e**), cytoplasm (**b, c**) and nuclear expression(**d**) through the immunohistochemical staining of breast cancer specimens (magnification, × 200)

The 283 breast cancer cases were then used to explore the relationship between β-catenin expression and clinicopathological parameters. Figure 2a represents the typical progression of breast cancer. It was observed that β-catenin presented a strong uniform membrane in the luminal cells of para-carcinoma normal tissues (Fig. 2a, left). In the high-grade ductal carcinoma in situ and invasive cancer, β-catenin demonstrated different expression patterns of cytoplasmic (Fig. 2a, middle) and nuclear expression (Fig. 2a, right). The different expression patterns of AJAP1 and β-catenin in different cases were illustrated in Fig. 2b–e. The abnormal membranous expression of β-catenin and the positive nuclear/cytoplasmic expression of β-catenin were detected in 43.5% (123/283) and 78.1% (221/283) of the breast cancer tissues, respectively. As shown in Table 2, the β-catenin expression in membrane staining was correlated with histological grade (*p* = 0.005) and lymph node (LN) involvement (*p* = 0.018) in breast cancer patients. Likewise, the expression of β-catenin in the nucleus and cytoplasm was related to histological grade (*p* < 0.0001) and LN involvement (*p* = 0.010) in breast cancer patients.

**Table 2:** Correlation between the clinicopathologic characteristics and membranous, cytoplasmic and nuclear expression of β-catenin in 283 breast cancer patients

Parameters	Cases	β-catenin(M)		*p*	β-catenin(C/N)		*p*
		Normal (N, %)	Abnormal (N, %)		Negative(N, %)	Positive (N, %)	
Age(yr)		160(56.5)	123(43.5)		62(21.9)	221(78.1)	
≤50	130	79(49.4)	51(41.5)	0.186	32(51.6)	123(55.7)	0.310
> 50	153	81(50.6)	72(58.5)		30(48.4)	98(44.3)	
Menopausal status							
Premenopausal	156	90(56.3)	66(53.7)	0.664	37(59.7)	119(53.8)	0.415
Postmenopausal	127	70(43.8)	57(46.3)		25(40.3)	102(46.2)	
Family history							
No	194	111(69.4)	83(67.5)	0.734	69(31.2)	152(68.8)	0.877
Yes	89	49(30.6)	40(32.5)		20(32.3)	69(31.2)	
Tumor size							
T1	110	66(41.3)	44(35.8)	0.428	32(51.6)	78(35.3)	0.055
T2	149	83(51.9)	66(53.7)		27(43.5)	122(55.2)	
T3	24	11(6.9)	13(10.6)		3(4.8)	21(9.5)	
Histological grade							
1	44	33(20.6)	11(8.9)	0.005*	16(25.8)	28(12.7)	0.029*
2	153	88(55.0)	65(52.8)		32(51.6)	121(54.8)	
3	86	39(24.4)	47(38.2)		14(22.6)	72(32.6)	
LN involvement							
0	180	114(71.3)	66(53.7)	0.018*	49(79.0)	131(59.3)	0.028*
1–3	43	19(11.9)	24(19.5)		7(11.3)	36(16.3)	
4–9	41	20(12.5)	21(17.1)		5(8.1)	36(16.3)	
≥ 10	19	7(4.4)	12(9.8)		1(1.6)	18(8.1)	
ER							
Negative	111	68(42.5)	43(35.0)	0.198	27(43.5)	84(38.0)	0.430
Positive	172	92(57.5)	80(65.0)		35(56.5)	137(62.0)	
PR							
Negative	142	87(54.4)	55(44.7)	0.107	35(56.5)	107(48.4)	0.263
Positive	141	73(45.6)	68(55.3)		27(43.5)	114(51.6)	
Her-2							
Negative	200	109(54.5)	91(45.5)	0.283	44(22.0)	156(78.0)	0.953
Positive	83	51(61.4)	32(38.6)		18 (21.7)	65 (78.3)	
Ki67							
< 20	83	54 (33.8)	29 (23.6)	0.062	19 (30.6)	64 (29.0)	0.797
≥ 20	200	106 (66.3)	94 (76.4)		43 (69.4)	157 (71.0)	
P53							
Negative	143	80(50.0)	63(51.2)	0.839	27(43.5)	116(52.5)	0.213
Positive	140	80(50.0)	60(48.8)		35(56.5)	105(47.5)	

*significantly different

There were different expression forms of AJAP1 and β-catenin observed in Fig. 2b–e, thus the correlation between the expressions of β-catenin and AJAP1 was analyzed. Table 3 showed that the low expression of AJAP1 was linked to abnormal membrane expression (*p* < 0.0001) and cytoplasmic/nuclear staining of β-catenin (*p* = 0.001). Moreover, the membrane staining of β-catenin had a significant difference with the cytoplasmic/nuclear expression (Table 4, *p* < 0.001).

**Table 3:** Correlation between β-catenin and AJAP1 expression

			AJAP1		*p*
			Low expression	High expression	
Membrane	160	Normal	49(41.5)	111(67.3)	< 0.0001*
	123	Abnormal	69(58.5)	54(32.7)	
Cytoplasmic/Nuclear	62	Negative	14(11.9)	48(29.1)	0.001*
	221	Positive	104(88.1)	117(70.9)

**Table 4:** Correlation between membrane and nuclear of β-catenin expression

			Cytoplasmic/Nuclear		*p*
			Negative	Positive	
Membrane	160	Normal	57(91.9)	103(46.6)	< 0.0001*
	123	Abnormal	5(8.1)	118(53.4)	

### Correlation between AJAP1 and β-catenin expressions with patient outcome

**Fig. 3:**
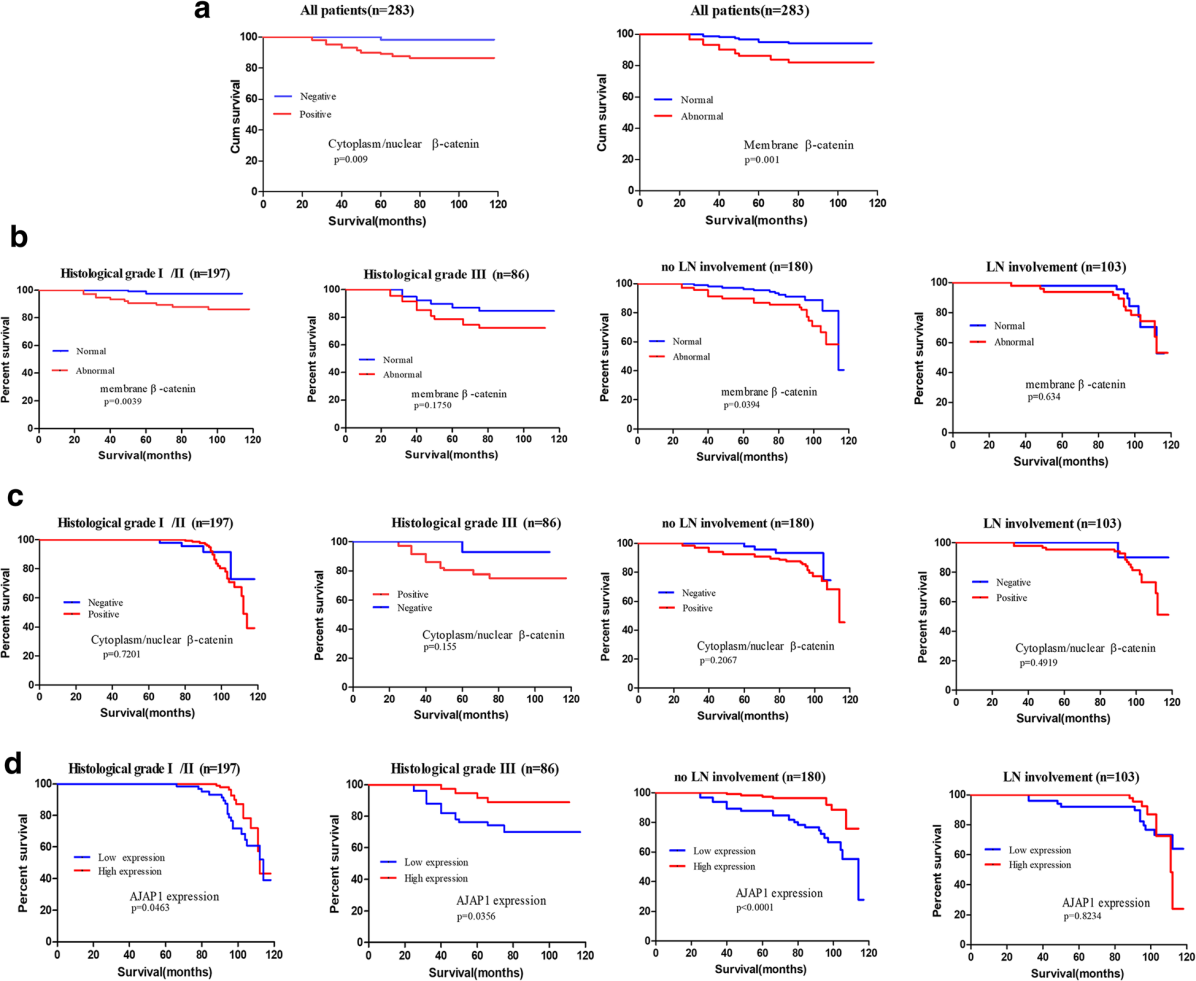
The effect of different expression locations of β-catenin and AJAP1 expression on overall survival was demonstrated in breast cancer patients. All breast cancer patients were divided into different groups according to histological grade and LN involvement. The Kaplan-Meier survival plot was evaluated, and log-rank tests were utilized to analyze the prognostic value of AJAP1 and β-catenin expressions in all patients (**a**) and subgroups (**b-d**)

As shown in Fig. 3a, abnormal membrane expression, cytoplasm and nuclear staining of β-catenin characterized a poor prognosis, while univariate and multivariate analyses showed that the location of β-catenin was not an independent prognostic factor for OS (Additional file 3: Table S3).

Many studies hold different perspectives on the prognosis value of β-catenin. The membrane expression of β-catenin seemed to predict OS better in the I/II histological grade (*p* = 0.0039) and no LN involvement (*p* = 0.0394) as shown in Fig. 3b. However, the cytoplasmic or nuclear expression of β-catenin did not show a significant difference in the different risks of subgroups in breast cancer patients (Fig. 3c).

Patients with a low expression of AJAP1 predicted poor OS in all the subgroups except the patients without involvement of LN (Fig. 3d, *p* = 0.8234). In summary, these results indicated that AJAP1 expression could be a prognostic marker in the different risks of subgroups of breast cancer patients, while β-catenin didn’t have the same function.

### AJAP1 inhibited nuclear accumulation of β-catenin via interaction with β-catenin binding sites in breast cancer cell

**Fig. 4:**
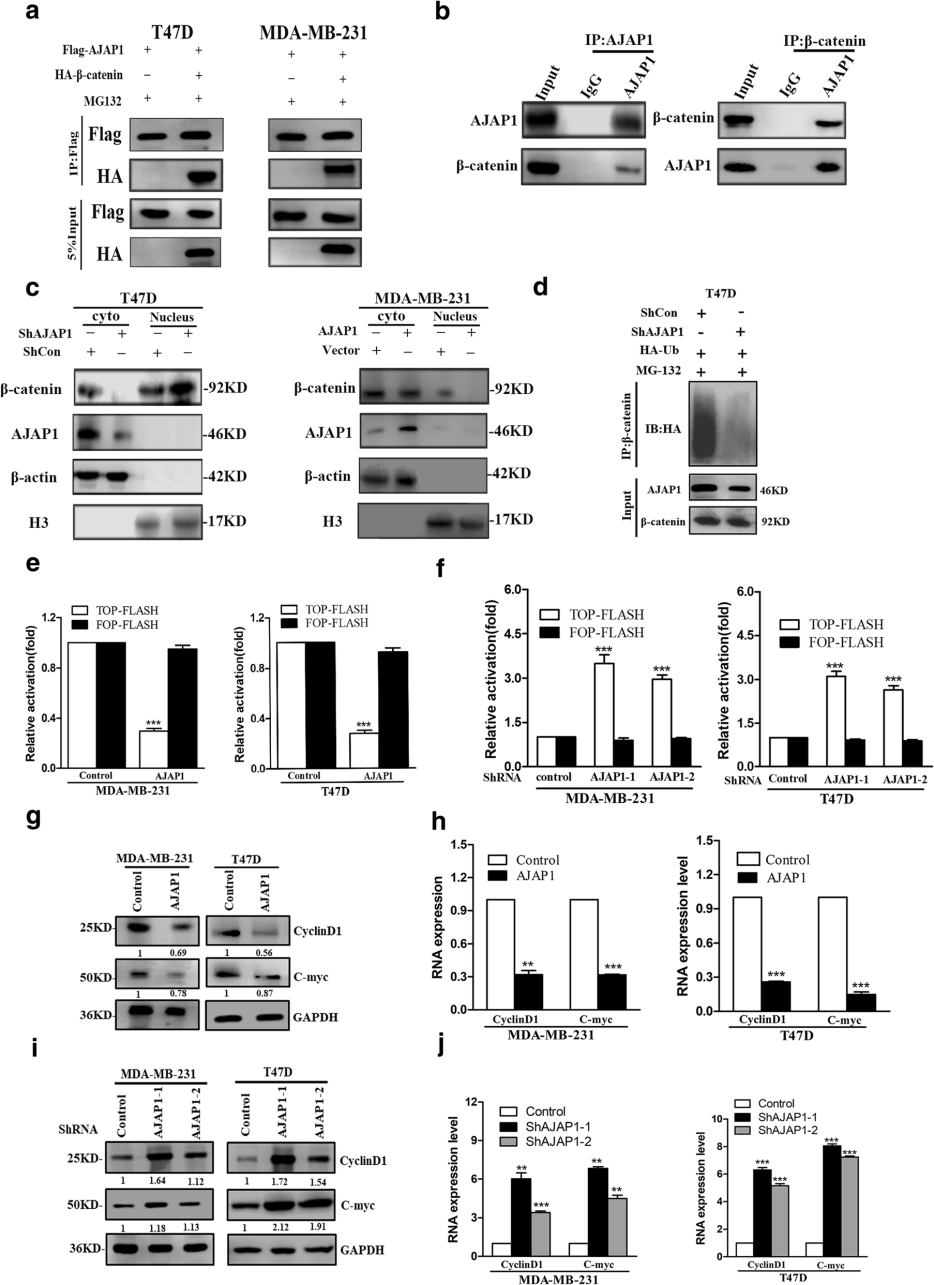
AJAP1 interacted with β-catenin and mediated its nuclear location and transcriptional activity. **a** Co-IP assay results showed that AJAP1 formed a complex with β-catenin in T47D and MDA-MB-231 cells. **b** Co-IP assays showed that endogenous protein of AJAP1 interacted with β-catenin. **c** Western blot results showed that AJAP1 depletion increased nuclear translocation of β-catenin and overexpression of AJAP1 had the opposite function. **d** AJAP1-depleted stable cell lines were transfected with HA-Ub and then treated with MG132 as demonstrated. Cell lysates were subjected to β-catenin and showed that AJAP1-depletion inhibited the ubiquitination level of β-catenin. **e** AJAP1 reduced the β-catenin/TCF/LEF-mediated transcription activity in MDA-MB-231 and T47D cells with and without the upregulation of AJAP1. **f** AJAP1-depletion in MDA-MB-231 and T47D cells increased the β-catenin/TCF/LEF-mediated transcription activity. **g-h** overexpressed AJAP1 can reduce the protein (**g)** and mRNA(**h**) levels of β-catenin downstream genes as C-myc and CyclinD1 in MDA-MB-231 and T47D cells by western blot and RT-PCR. **i-j** AJAP1 knockdown can increase the protein (**i**) and mRNA(**j**) levels of β-catenin downstream genes, such as C-myc and CyclinD1 in MDA-MB-231 and T47D cells by western blot and qRT-PCR. Data were shown as mean ± SD. Each experiment was conducted in triplicate. **p < 0.01, ***p < 0.001

To further explore how AJAP1 mediated β-catenin, the relationship and potential mechanism between AJAP1 and the different locations of β-catenin in breast cancer were investigated. Firstly, it was found that AJAP1 and β-catenin interacted in T47D and MDA-MB-231 cells (Fig. 4a). Co-IP assay was also conducted to confirm this theory (Fig. 4b). Loss of AJAP1 reduced the β-catenin cytoplasm expression but increased its nuclear location in T47D. In contrast, upregulated AJAP1 in MDA-MB-231 cells showed opposite results (Fig. 4c). To determine the exact path of β-catenin degradation mediated by AJAP1, the ubiquitination level of β-catenin in AJAP1-deficient T47D cells was examined. As shown in Fig. 4d, the ubiquitination of β-catenin was reduced in the AJAP1 knockdown cells, demonstrating that AJAP1-depletion activated Wnt signaling which caused β-catenin degradation.

### AJAP1 regulated the transcriptional activity of β-catenin and its downstream gene expression

Above experiments showed that the knockdown of AJAP1 influenced the location of β-catenin, however it is unclear whether the transcriptional activity of β-catenin was also changed. TCF/LEF-responsive luciferase (TOP-FlASH) and non-responsive (FOP-FLASH) reporters were used to evaluate the effect of AJAP1 on the β-catenin transcriptional activity. As shown in Fig. 4e, upregulation of AJAP1 led to a lower transcription activity from the β-catenin/TCF/LEF-dependent promoter (TOP-FLASH) but had little effect on FOP-FLASH. Conversely, downregulation of AJAP1 obviously increased the transcription activity of the β-catenin/TCF/LEF-dependent promoter (Fig. 4f). To further examine the influence of knockdown or overexpression of AJAP1 on β-catenin’s downstream gene expression, the expressions of C-myc and CyclinD1 were analyzed by Western blotting and qRT-PCR. Overexpression of AJAP1 in MDA-MB-231 and T47D cells decreased C-myc and CyclinD1 expression both in protein and mRNA levels (Fig. 4g and h). On the contrary, knockdown of AJAP1 showed the opposite results (Fig. 4i and j). These findings showed that AJAP1 depletion increased β-catenin nuclear expression, activated the transcriptional activity of β-catenin and promoted its expressions of downstream genes (C-myc and CyclinD1).

### AJAP1 regulated breast cancer tumorigenesis via mediating β-catenin expression

**Fig. 5:**
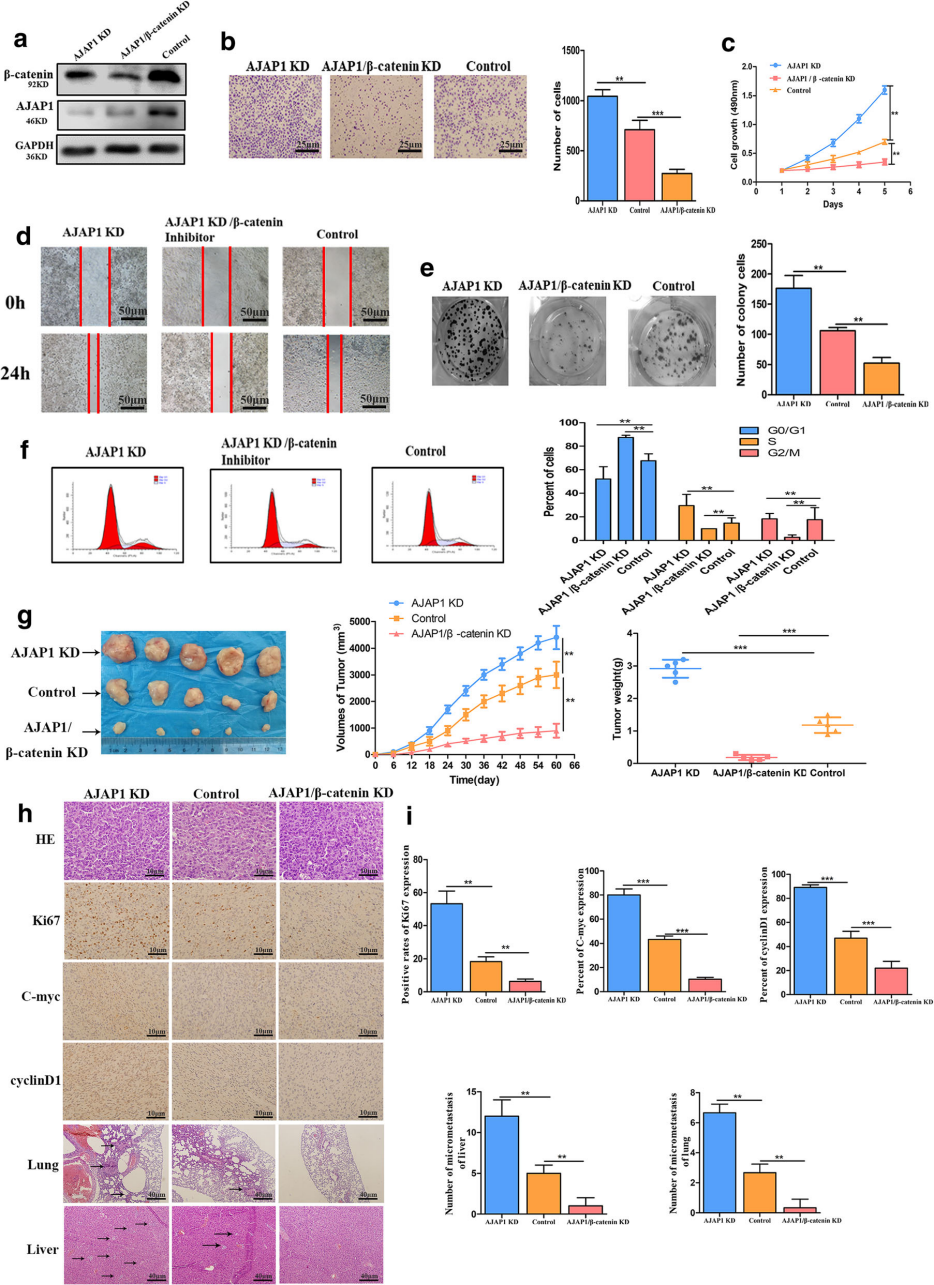
AJAP1 regulated tumor progression by regulating β-catenin in breast cancer **a** Validation of protein levels of three groups after transfected with related shRNA by Western Blot **b、d** Results of the transwell assay (**b**) and wound-healing assay(**d**) showed that AJAP1/β-catenin KD inhibited invasion and migration abilities of T47D cell, indicating that β-catenin can reverse the effect of AJAP1-depletion caused (Transwell assay: original magnification, × 200). **c、e** MTT(**c**) and Colony-forming assay(**e**) were conducted to determine the clone-initiating and proliferation ability among AJAP1 KD, AJAP1/β-catenin KD and control in T47D cells. **f** Flow cytometry results of AJAP1 KD, AJAP1/β-catenin KD and control in T47D cells. **g** Representative images of tumor isolated from three groups (left panel) as demonstrated and tumor growth (middle panel), tumor weight representative graph(right) **h** H&E staining of tumors isolated from three experimental groups (magnification, × 400), IHC staining of Ki67, C-myc and CyclinD1 in slides removed from the three groups (magnification, × 400) and typical images of micrometastasis of the lung and liver (magnification, × 100). **i** Corresponding analysis of the positive rates of Ki67, C-myc and CyclinD1 expression in three groups(top), the number of liver and lung micrometastasis in three related groups. Data were shown as mean ± SD. Each experiment was conducted in triplicate. **p < 0.01, ***p < 0.001

The above results indicated that AJAP1 inhibited cell invasion, proliferation and migration ability in breast cancer. Then we explored whether the function of AJAP1 in breast cancer progression relied on mediation of β-catenin expression. For this purpose, three groups of T47D cells were constructed: depletion of AJAP1 (AJAP1 KD), AJAP1 knockdown and β-catenin knockdown (AJAP1/β-catenin KD), and the control. Figure 5a shows the different protein levels of AJAP1 and β-catenin in the three groups. Transwell assay and wound healing were used to examine the migratory and invasion abilities of these cells. As shown in Fig. 5b and Fig. 5d, AJAP1/β-catenin KD group suppressed the migratory and invasion abilities in comparison with the AJAP1 KD and control groups. Colony formation and MTT assays were performed on the three groups and the results showed that the proliferation ability of the AJAP1/β-catenin KD group was also lower than AJAP1 KD and control groups (Fig. 5c and Fig. 5e). The cell cycle results demonstrated that the AJAP1/β-catenin KD group had a higher number of G0/G1 cells but a lower number of S/G2/M cells than the other two groups (Fig. 5f). This phenomenon indicated that inhibited β-catenin expression reversed the effect caused by the depletion of AJAP1.To better analyze the significance of β-catenin in the tumorigenicity of AJAP1-mediated tumor progression, stable T47D cells were successfully established in the AJAP1 KD, AJAP1/β-catenin KD, and control groups. The AJAP1/β-catenin KD group had the lowest tumor capacity and tumor growth rate among the three groups (Fig. 5g).

The expression levels of ki67, C-myc, CyclinD1, lung and liver metastasis situation were evaluated in the mouse xenografts (Fig. 5h and i). It is clear that AJAP1/β-catenin KD group shows the lower expression of Ki67, less metastasis in lung and liver compared with the other two groups. This group also shows the lowest C-myc and CyclinD1 expressions (Fig. 5h and i). Collectively, AJAP1 inhibited breast cancer growth by regulating β-catenin transcriptional activity through suppressing C-myc and CyclinD1 expression both in vivo and in vitro.

### β-Catenin nuclear localization negatively mediated by EGF/EGFR attenuate AJAP1 expression

**Fig. 6:**
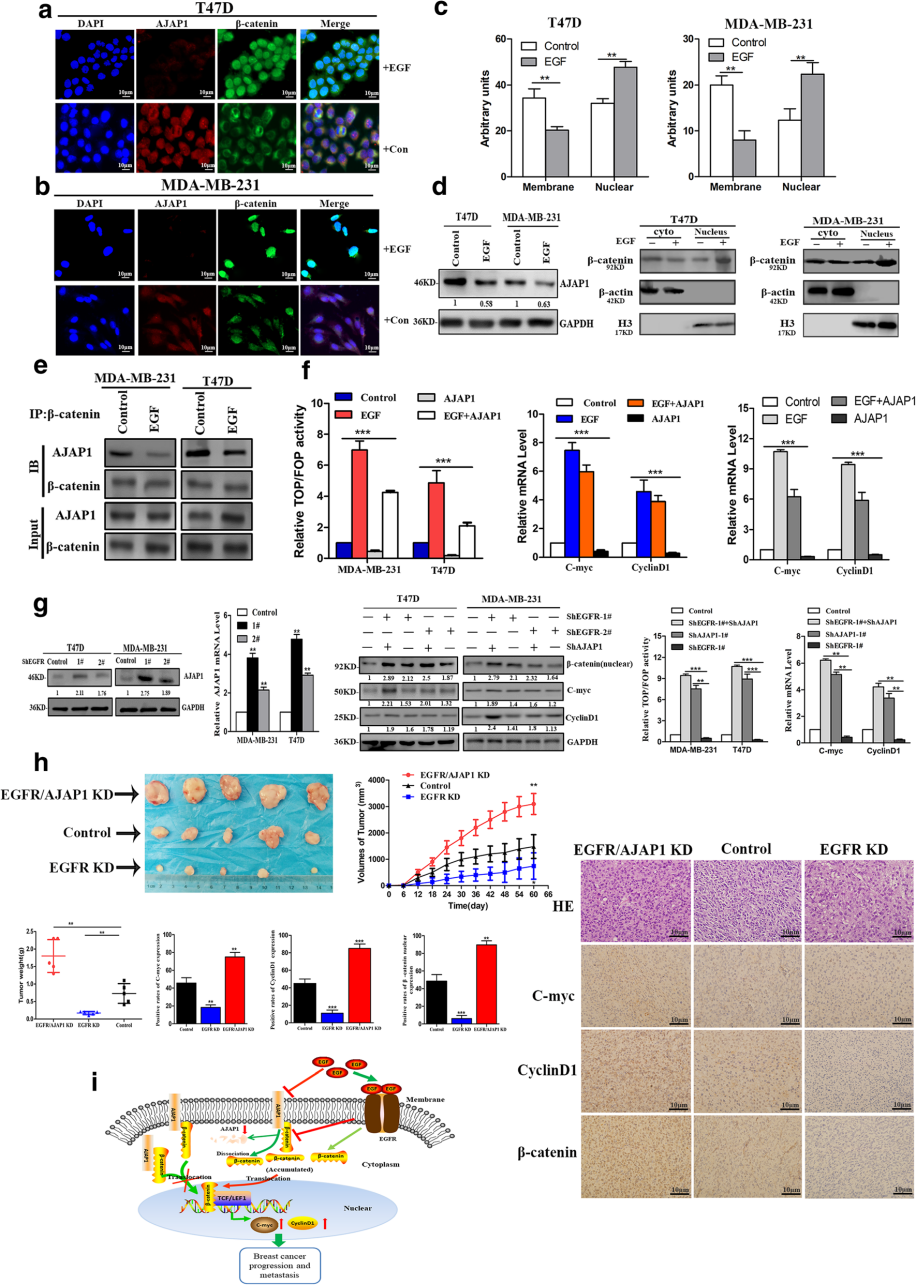
EGF and EGFR regulated the AJAP1-mediated β-catenin nuclear translocation. **a-b** The changes of AJAP1 and β-catenin were observed through immunofluorescence assay of both cells with EGF stimulation (100 ng/ml). (magnification, × 400) **c** Statistical analysis of the membrane and nuclear expressions of β-catenin with EGF treatment by Image J. **d** protein changes of AJAP1 and β-catenin expression in T47D and MDA-MB-231 cells under EGF stimulation **e** Immunoprecipitation assays were conducted to evaluate the interaction between AJAP1 and β-catenin under EGF treatment. **f** Relative luciferase activities of TOP/FOP dual-luciferase reporter performed to observe the effects of EGF (100 ng/ml) on both cell lines (left) qRT-PCR was performed to assess the effects of EGF stimulation on the β-catenin downstream genes C-myc and CyclinD1 in the experimental groups. **g** Western blot and qRT-PCR were conducted to examine the AJAP1 expression in EGFR-depleted without EGF stimulating cells (first and second panel). The results showed that EGFR-depletion in AJAP1 knocked down cells promote the β-catenin transfer to the nucleus and its transcription activity (third and fourth panels) and increased the downstream gene levels (third and last panels). **h** the results of tumor growth, H&E and immunohistochemistry staining of C-myc,CyclinD1, and β-catenin nuclear expression in vivo assays (magnification, × 400). Data were expressed as mean ± SD. **p < 0.01, ***p < 0.001 **i** A mechanism model showed that the β-Catenin nuclear localization positively fed back on the EGF/EGFR attenuated AJAP1 expression in breast cancer to promote breast cancer progression and metastasis

The above experiments verified β-catenin as the downstream target of AJAP1 in controlling breast cancer tumorigenesis and metastasis. However, it is necessary to further investigate which molecule controls the AJAP1 expression or mediate the conjunction between AJAP1 and β-catenin. Using the String online prediction software and the reported literature (Additional file 4: Figure S1), it was presumed that EGFR or EGF could participate in the AJAP1-β-catenin axis modulation process. To verify this assumption, EGF was added to T47D and MDA-MB-231 cells to observe the locational changes of β-catenin and AJAP1 through immunofluorescence assay**.** Consistently, Fig. 6a-c showed that EGF led the β-catenin expression transfer from the membrane/cytoplasm to the nucleus. Besides, the expression of AJAP1 was reduced but didn’t show locational changes. Western blot validated the same results in the MDA-MB-231 and T47D cell lines (Fig. 6d). In the subsequent co-IP assay, Fig. 6e presented that interaction between AJAP1 and β-catenin was obviously deceased by EGF. However, the total β-catenin of the cells did not change significantly. These experiments confirmed that β-catenin transferred from the cytoplasm to the nucleus. Subsequently, transcriptional activity of β-catenin and its downstream genes, such as C-myc and CyclinD1, were explored via luciferase assay and qRT-PCR. Figure 6f shows that the overexpression of AJAP1 with EGF reduced the relative TOP/FOP activity and the C-myc 、CyclinD1 mRNA levels in comparison with the group treated EGF alone. Taken together, EGF promoted the transfer of β-catenin from the membrane/cytoplasm to the nucleus and activated its transcriptional activity by inhibiting the expression of AJAP1.

EGF is considered as a ligand part of EGFR, and previous studies have demonstrated that its activation could stabilize and enhance the β-catenin nuclear accumulation in cancer [32–34]. Thus, investigations were carried out on whether EGFR had the same function as EGF and whether it participated in the process of AJAP1-mediated β-catenin expression. Cells without EGF stimulation were used to observe above changes. As shown in the first and second panels of Fig. 6g, EGFR depletion obviously inhibited the expression of AJAP1, reduced the expression of β-catenin and its downstream genes (C-myc and CyclinD1). However, knockdown AJAP1 and EGFR group also enhanced these effects and increased the relative β-catenin TOP/FOP activity.

Overall, these results showed that EGFR and EGF could inhibit the expression of AJAP1 and promot the β-catenin nuclear localization in the breast cancer cell lines.

### AJAP1 knockdown inhibited tumorigenicity of EGFR-depletion groups in vivo

Numerous reports showed that overexpression of EGFR decreased OS and DFS in women with early breast cancer [35, 36]. Therefore, it also seemed to be a major driving factor of malignancy in breast cancer [37–39]. Experiments were carried out to study whether AJAP1 had the same effect on EGFR in vivo. As shown in Fig. 6h, AJAP1/EGFR KD group improved the tumor growth and weight, β-catenin nuclear expression and the expressions of C-myc and CyclinD1 compared with the other two groups isolated from the breast cancer cell-inoculated nude mice.

Figure 6i showed that AJAP1 could interact with β-catenin and the loss of AJAP1 could promote breast cancer progression and metastasis by stimulating β-catenin nuclear translocation and TCF/LEF1 transcriptional activity, increasing C-myc and CyclinD1 expression. Furthermore, EGF/EGFR could inhibit the expression of AJAP1 and negatively control the location and the activity of β-catenin. EGF reduced the AJAP1–β-catenin complex joint efficiency by reducing AJAP1 expression and dissociating β-catenin from the junctions. Further, dissociative β-catenin could accumulate in the cytoplasm and then move to the nucleus, which aggravates the breast cancer malignancy. Collectively, EGF/EGFR/AJAP1 axis controled breast cancer tumor progression and metastasis by mediating the β-catenin nuclear transactivation.

## Discussion

Adherens junction (AJs) are multi-protein complexes which play vital roles in many behaviors of the cell like adhesion, transfer signals for inhibition of cell growth, resistance to apoptosis and regulation of cell shape and polarity [40–42]. Besides, AJs core structural component is a complex of cadherin and catenin proteins [40]. β-catenin can bind to the cadherins in AJs. The novel molecule of AJs, AJAP1, was found to be a tumor suppressor in glioma, hepatocellular carcinoma, esophagus carcinoma, oligodendrogliomas, and cervical cancer [12–14, 17, 43–45]. This study demonstrated that AJAP1 was a putative suppressor in breast cancer and it can interact with β-catenin in the cytoplasm and membrane. AJAP1 knocked down contributed to breast cancer malignancy through promoting accumulation of aberrant β-catenin nuclear expression, which indicated that increased expression of AJAP1 in higher grades of breast cancer could be beneficial to abrogate the β-catenin driven malignancy. Besides, the results of AJAP1 and β-catenin expression in 283 cases of breast cancer patients and overall survival further confirmed this theory.

As for β-catenin stabilization, recent researches [32, 34, 46, 47] have revealed that the accumulation of β-catenin nuclear expression activated many oncogenes about cellular proliferation. It is noteworthy that β-catenin is an important molecule which is involved in many metastasis signals. Thus, inhibiting nuclear accumulation of β-catenin might be an effective strategy for prohibiting malignancy of tumor cells. Here, a new mechanism for β-catenin nuclear location was identified. AJAP1 depletion promoted β-catenin nuclear translocation, affected the transcription activity of β-catenin and its downstream genes like C-myc and CyclinD1 expression. Likewise, AJAP1 regulated breast cancer tumorigenesis via mediating the β-catenin activity, indicating an effective way to prevent the tumor progression.

Increasing evidence demonstrated that EGF affected the β-catenin transactivation in breast cancer [33, 46, 48]. As expected, it was found that EGF-stimulated cells accelerated β-catenin nuclear transaction, decreased AJAP1 expression and activated the C-myc and CycliD1 expression. Previous studies revealed that EGF activated EGFR and led to the activation of downstream signaling pathways, such as PI3K/AKT and MAPK/ERK, which are critical in metastasis and tumor progression [49–51]. And now EGFR mutant also occurred in breast cancer. A great deal of studies show that EGFR-target medicine made a huge breakthrough in treating cancer especially lung cancer. Above achievements inspired us to use these target medicine to treat breast cancer with high EGFR mutant rates. Besides, our results showed that EGFR had similar function on β-catenin as previously illustrated [32, 34]. Additionally, Chao Yang et al. [13] revealed that EGFR/EGFRvIII inhibited AJAP1 in the cytoskeleton remodel of glioma cell. However, our study explored and validated that EGFR also can inhibit AJAP1 expression and β-catenin activity in breast cancer cells. Knockdown of EGFR and AJAP1 promoted tumor growth and β-catenin nuclear expression. Collectively, EGFR acted as an important role activating β-catenin nuclear expression. These results implied that the combination of EGFR targets and AJAP1-enhanced medicine might improve the prognosis of breast cancer patients. However, further researches on different β-catenin phosphorylation spots and mutants mediated by AJAP1 need to be further conducted. Likewise, these discoveries inspire more studies to explore the role of AJAP1 in EGFR-target medicine like Gefitinib in future studies.

To the best of our knowledge, this is the first study to reveal that the aberrant nuclear localization of β-catenin in breast cancer tissues, which is associated with the expression level of AJAP1. This work clarified one new feed loop for EGF-EGFR-AJAP1-controlled nuclear location and transcription activity of β-catenin. This change affected breast cancer progression and metastasis. Given the inhibition effect of AJAP1 on breast cancer both in vivo and in vitro, new medicine targets need to be developed. Determining whether this inducer for AJAP1 has the same effect as other adherens junction-associated proteins is also significant. If so, then many new anticancer occurrences may bring new hope to breast cancer patients.

## Conclusion

We reported a novel EGF/EGFR axis that negatively fed back on the AJAP1-mediated β-catenin expression pathway and it accelerated breast cancer progression. This research provided novel findings demonstrating that the combination of the inhibitor of EGFR and the AJAP1 inducer may be beneficial to breast cancer prognosis.

## Additional files

Additional file 1: **Table S1.** Clinicopathological parameters of breast cancer patients. (DOC 54 kb)

Additional file 2: **Table S2.** Primers of real-time PCR and oligos for siRNAs or shRNAs in this study. (XLSX 9 kb)

Additional file 3 **Table S3.** Cox proportional hazard regression model analysis. (DOC 36 kb)

Additional file 4: **Figure S1.** Predicted molecules that may be associated with AJAP1 and β-catenin using STRING software. (TIF 3124 kb)

## Data Availability

The datasets used and analyzed during the current study are available from the corresponding author on reasonable request.

## Abbreviations

AJAP1: Adherens junctions associated protein 1
AJs: Adherent junctions
EGF: Epidermal Growth Factor
EGFR: Epidermal Growth Factor Receptor
LN: Lymph node
qRT-PCR: quantitative reverse-transcription polymerase chain reaction
