# Supplementary material for: β-Catenin nuclear localization positively feeds back on EGF/EGFR-attenuated AJAP1 expression in breast cancer
Source: J Exp Clin Cancer Res. 2019 Jun 6;38:238. doi: 10.1186/s13046-019-1252-6 (PMC6554977; doi:10.1186/s13046-019-1252-6)
Supplement: Supplementary file 1 — Table S1. Clinicopathological parameters of breast cancer patients. (DOC 54 kb) [file 13046_2019_1252_MOESM1_ESM.doc]

**Additional file 1: Table S1 Clinicopathological parameters of breast cancer patients**

| **Parameters** | **No** | **%** |
| --- | --- | --- |
| **Age(years)** |  |  |
| **≤50** | 130 | 45.9 |
| **>50** | 153 | 54.1 |
| **Menopausal status** |  |  |
| Premenopausal | 156 | 55.1 |
| Postmenopausal | 127 | 44.9 |
| **Family history** |  |  |
| No | 194 | 68.6 |
| Yes | 89 | 31.4 |
| **Tumor Size** |  |  |
| T1 | 110 | 38.9 |
| T2 | 149 | 52.7 |
| T3 | 24 | 8.5 |
| **Histological grade** |  |  |
| 1 | 44 | 15.5 |
| 2 | 153 | 54.1 |
| 3 | 86 | 30.4 |
| **LN involvement** |  |  |
| 0 | 180 | 63.6 |
| 1-3 | 43 | 15.2 |
| 4-9 | 41 | 14.5 |
| ≥10 | 19 | 6.7 |
| **ER** |  |  |
| Negative | 111 | 39.2 |
| Positive | 172 | 60.8 |
| **PR** |  |  |
| Negative | 142 | 50.2 |
| Positive | 141 | 49.8 |
| **Her-2** |  |  |
| Negative | 200 | 70.7 |
| Positive | 83 | 29.3 |
| **Ki67** |  |  |
| <20 | 79 | 27.9 |
| ≥20 | 204 | 72.1 |
| **P53** |  |  |
| Negative | 143 | 50.5 |
| Positive | 140 | 49.5 |
| **Chemotherapy** |  |  |
| No | 119 | 42.0 |
| Yes | 164 | 58.0 |
| **Radiotherapy** |  |  |
| **No** | 219 | 77.4 |
| **Yes** | 64 | 22.6 |
| **Endocrine** |  |  |
| **No** | 118 | 41.7 |
| **Yes** | 165 | 58.3 |
