# Supplementary material for: β-Catenin nuclear localization positively feeds back on EGF/EGFR-attenuated AJAP1 expression in breast cancer
Source: J Exp Clin Cancer Res. 2019 Jun 6;38:238. doi: 10.1186/s13046-019-1252-6 (PMC6554977; doi:10.1186/s13046-019-1252-6)
Supplement: Supplementary file 3 — Table S3. Cox proportional hazard regression model analysis. (DOC 36 kb) [file 13046_2019_1252_MOESM3_ESM.doc]

**Additional file 3: Table S3. Cox proportional hazard regression model analysis**

| **Variables** | **Univariate analysis** | | ***p* value** | **Multivariate analysis** | | ***p* value** |
| --- | --- | --- | --- | --- | --- | --- |
|  | **HR** | **95%CI** |  | **HR** | **95%CI** |  |
| **Age (≤50y vs> 50y)** | 1.453 | 0.716-2.948 | 0.300 |  |  |  |
| **Menopausal status  (premenopausal vs. Postmenopausal)** | 0.487 | 0.224-1.057 | 0.069 |  |  |  |
| **Family history（No vs. Yes)** | 1.967 | 0.807-4.794 | 0.137 |  |  |  |
| **Tumor size (T1 vs. T2、T3)** | 2.267 | 0.977-5.262 | 0.057 |  |  |  |
| **Histological grade(1 vs.2 、.3)** | 26.479 | 0.430-1631.673 | 0.119 |  |  |  |
| **LN involvement**  **(negative vs. positive)** | 0.974 | 0.467-2.032 | 0.944 |  |  |  |
| **AJAP1**  **(Low expression VS High expression)** | 0.318 | 0.150-0.675 | 0.003^*^ | 0.411 | 0.190-0.889 | 0.024^*^ |
| **β-catenin(M)**  **(Normal vs Abnormal)** | 3.396 | 1.563-7.376 | 0.002^*^ | 2.222 | 0.992-4.980 | 0.052 |
| **β-catenin(C/N)**  **(Negative/Positive)** | 8.843 | 1.206-64.844 | 0.032* | 5.251 | 0.683-40.388 | 0.1111 |
