# Supplementary figures and images for: β-Catenin nuclear localization positively feeds back on EGF/EGFR-attenuated AJAP1 expression in breast cancer
Source: J Exp Clin Cancer Res. 2019 Jun 6;38:238. doi: 10.1186/s13046-019-1252-6 (PMC6554977; doi:10.1186/s13046-019-1252-6)

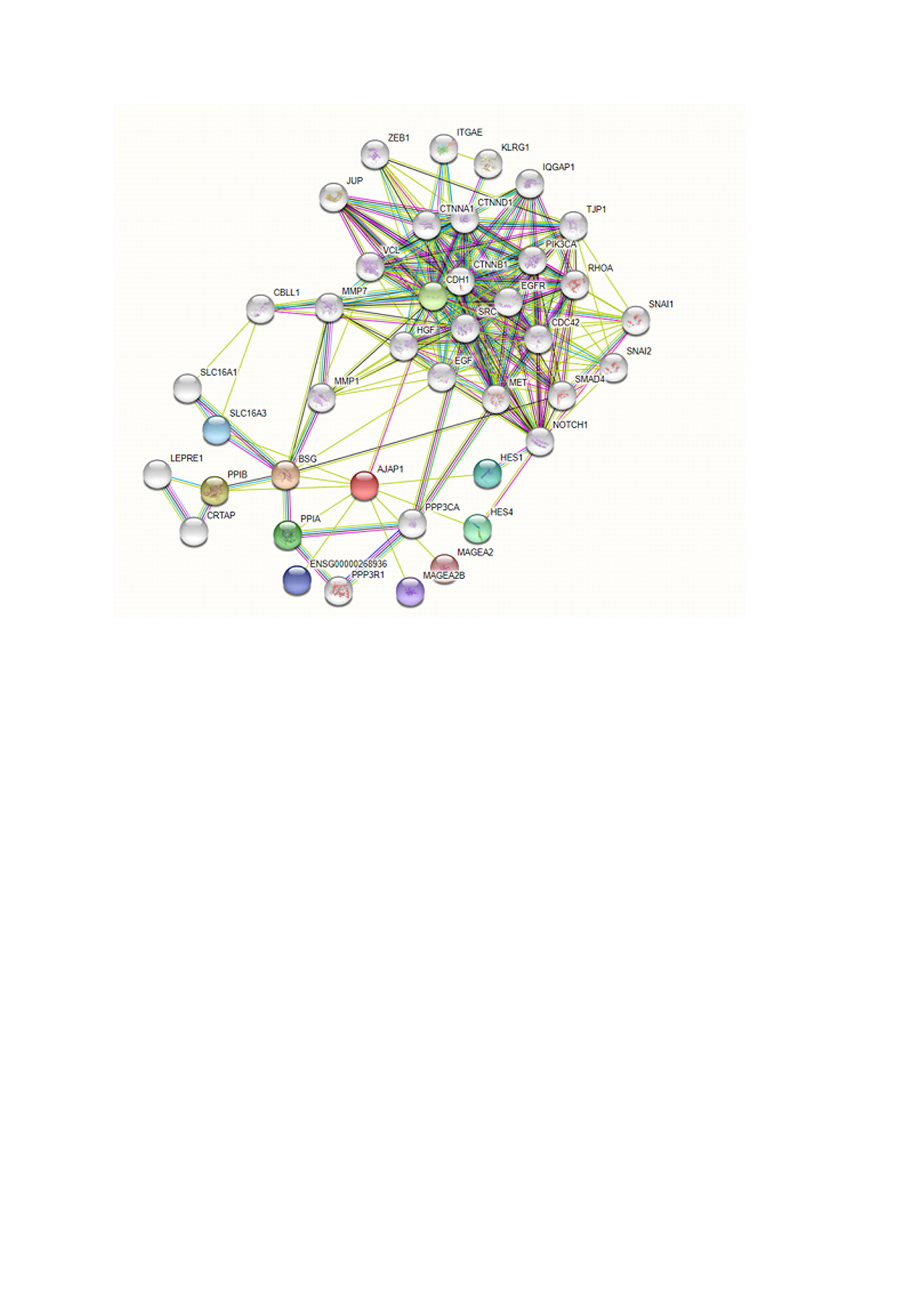

Supplement: Supplementary file 4 — Figure S1. Predicted molecules that may be associated with AJAP1 and β-catenin using STRING software. (TIF 3124 kb) [file 13046_2019_1252_MOESM4_ESM.tif]
